# Revision Hip Arthroplasty in Patient with Acetabulum Migration into Subperitoneal Space—A Case Report

**DOI:** 10.3390/medicina57010030

**Published:** 2020-12-31

**Authors:** Andrzej Kotela, Jacek Lorkowski, Dariusz Chmielewski, Marta Grodzik, Ireneusz Kotela

**Affiliations:** 1 Faculty of Medicine, Collegium Medicum, Cardinal Stefan Wyszynski University in Warsaw, Woycickiego 1/3, 01-938 Warsaw, Poland; 2 Department of Orthopedic Surgery and Traumatology, Central Research Hospital of Ministry of Interior, Wołoska 137, 02-507 Warszawa, Poland; jacek.lorkowski@gmail.com (J.L.); ikotela@op.pl (I.K.); 3 Department of Orthopaedic Surgery and Traumatology, HOSPITEN, Lomo Gordo s/n, 35500 Puerto del Carmen/Lanzarote, Spain; dariusz.chmielewski@hospiten.com; 4 Institute of Biology, Warsaw University of Life Sciences, Ciszewskiego 8, 02-786 Warsaw, Poland; 5 Department of Interventional Medicine with the Laboratory of Medical Genetics, Institute of Medical Sciences, Collegium Medicum, Jan Kochanowski University, IX Wiekow 19, 25-317 Kielce, Poland

**Keywords:** revision arthroplasty, hip joint, acetabular migration, protrusion, Paprosky Type III B acetabular defect, subperitoneal space

## Abstract

Revision hip arthroplasty procedures have been extensively discussed in the literature. At the same time, discussions of the management of acetabular component protrusion into the pelvic cavity, and, more specifically, the subperitoneal space, necessitating an additional abdominal approach for the revision arthroplasty, have only been published as case reports and descriptions of transperitoneal approaches have been even rarer. This paper presents the case of a 63-year-old female patient in whom a peritoneal approach was necessary to access a migrated acetabular component. The outcome of the treatment, which represented a complex orthopedic and general surgical problem, was good. We believe that the complexity of revision hip arthroplasty in patients with protrusion of the acetabular component together with the head and proximal part of the stem of the implant into subperitoneal space calls for a careful re-analysis of the category of Type III bony acetabulum defects according to Paprosky, where the recognition of two subtypes would facilitate analysis of such cases.

## 1. Introduction

Revision arthroplasty for a loosened hip endoprosthesis has become a routine procedure performed by orthopedic surgeons. Although the surgical skills and technical possibilities are constantly improving, this specific intervention exposes the patient to a number of risks, like neurovascular damage and pelvic organs lesion. Revision hip arthroplasty has been extensively described in the literature [1,2,3,4,5,6,7]. Cases of bottom of the acetabulum damage and migration or accidental displacement of the implanted socket into the pelvic cavity represent a more complex problem requiring more than standard management. Such cases have been described in important, but isolated, reports. The correct and effective management of such patients requires co-operation between orthopedic and general surgical team [8,9,10]. Safe removal of the intrapelvic material seems to be based on some principles: detailed preoperative analysis with identification of potential risk, planning and scheduling of surgical procedure especially focused on preserving of important organs and tissues, saving bone stock, restoring biomechanics of pelvis en hip (including correction of any length discrepancy) [10]. This report describes the diagnostic work-up, treatment and long-term treatment outcomes in a case of migration of the acetabular component of an implant into the pelvic cavity, and, more specifically, the subperitoneal space [11]. Access to the loosened implant had to be gained via the peritoneal cavity [12]. This case report appears worthwhile not only because such problems are rare in the clinical practice, but also in view of the good long-term clinical outcome.

## 2. Case Report

**Figure 1:**
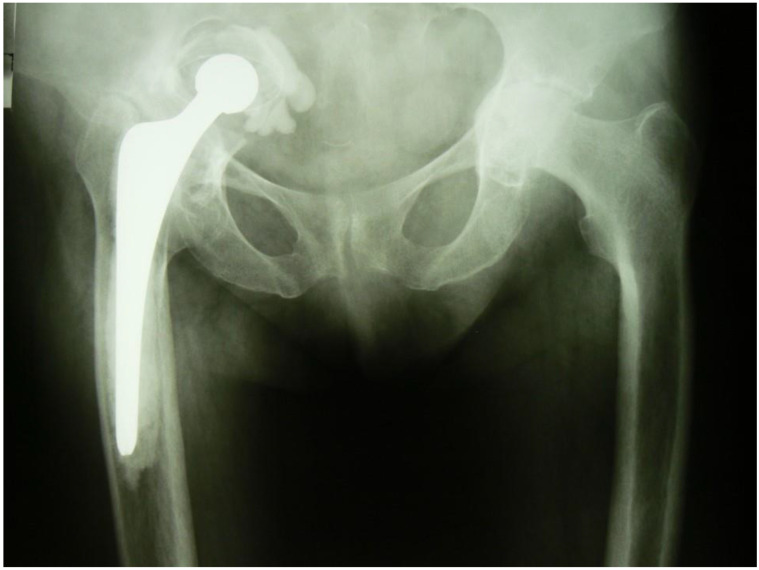
Protrusion of the prosthetic acetabulum, migration of the acetabular component, head and proximal part of the stem above the iliopectineal line. An antero-posterior (AP) radiograph of the pelvis and the hip joints.

G.M., a female aged 63 years, was admitted to the Department on account of loosening of the acetabulum of the right hip joint with damage to the roof of the bony acetabulum and migration of the implant’s acetabular component into the pelvic cavity, and more specifically, into subperitoneal space (Figure 1). The patient used a wheelchair for mobility. There was trace movement in the right hip. Assessment was difficult because of intense pain on attempting to move the hip. The HHS (Harris Hip Score) was 15.7 and the NRS (Numeric Rating Scale) for patient self-reporting pain severity was 6.

The history and available medical documentation revealed that the patient had undergone a total cemented arthroplasty (Ultima, Johnson&Johnson) of the right hip joint at another center two years earlier. The indication was secondary osteoarthritis of the hip in the course of Otto-Chrobak disease [13,14,15]. The patient reported that the outcome of the treatment had been good for 18 months following the primary arthroplasty procedure. At that time, without suffering a distinct injury, the patient felt severe pain in her right hip joint, a cracking noise in the joint and then she could not move about. Any attempts at moving the joint caused intense pain. She was diagnosed with protrusion of the implant acetabulum into the abdominal cavity and referred to our center after a few weeks.

Before the revision surgery, the patient underwent a careful diagnostic work-up in accordance with recommendations from the literature and our own experience.

A computed tomography (CT) can of the hip joint showed destruction of the pelvic acetabulum of Type III B according to Paprosky [16]. The implant’s acetabular component, head and proximal part of the stem were also seen displaced into the pelvic cavity. The images were difficult to interpret in view of numerous artifacts associated with the presence of large amounts of metal in the area.

An angio–CT study revealed a patent right common iliac artery, internal and external iliac arteries, femoral artery and the deep artery of the hip with minor atherosclerotic areas of stenosis. The right external iliac artery in its proximal segment had a diameter of 12 mm and was 25 mm away from the intraabdominally displaced acetabular component of the implant.

An abdominal ultrasound exam did not reveal significant pathology of the internal organs, including specifically those organs adjacent to the displaced acetabular component. There were no signs of compression of the right ureter.

Consultations with a general surgeon, vascular surgeon, gynecologist, and urologist were arranged. The consulting specialists were prepared to undertake an intraoperative intervention if that should turn out to be necessary. The gynecological and urological consultations also confirmed the absence of pathology within the respective systems.

## 3. Results

### Report of the Operative Procedure

**Figure 2:**
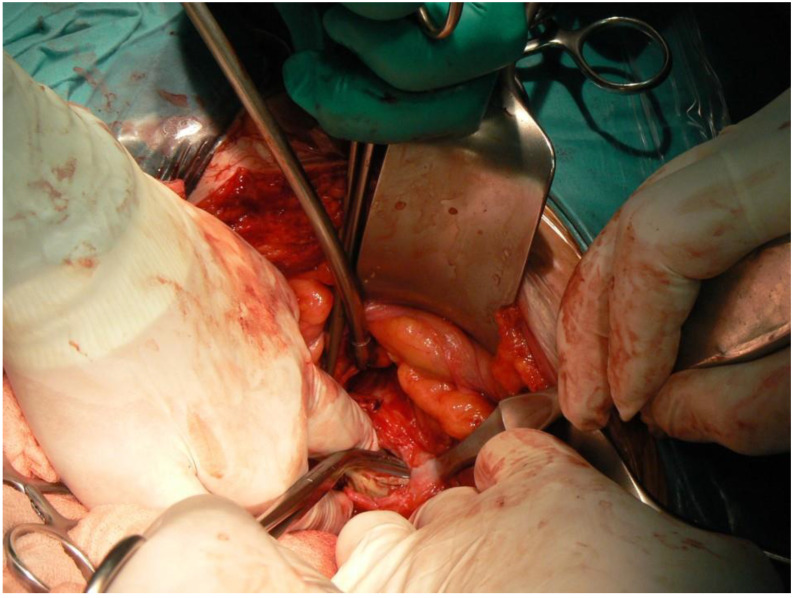
Removal of the acetabular component and bone cement from the pelvic cavity—intraoperative photograph.

An antero-lateral incision (Watson-Jones) in supine position on the flat orthopedic operation table was used to expose the right hip joint. Displacement of the implant’s acetabular component, head, and proximal part of the stem into the pelvic cavity was confirmed. Specimens for histology and culture were obtained. It was not possible to safely remove the implant by approaching it via the lower limb. Attempts to reduce the centrally dislocated implant from subperitoneal space back to its normal anatomic location were futile both when traction was used and when dislocation was attempted. Consequently, the general surgery team was requested to open the abdominal cavity. The surgical access in the right hypogastrium was by a typical oblique incision parallel to the inguinal ligament. The posterior abdominal wall was reached via the peritoneal cavity. The inferior abdominal wall revealed bulging of the iliac muscle, covered with parietal peritoneum. The ureter was slightly (approximately 1.5 cm) displaced medially but was not compressed. The right iliac muscle was dissected over the displaced iliac acetabulum. Signs of partial damage to the muscle over the implant acetabulum and the bulging of cement directly below the parietal peritoneum were found. The acetabular component with bone cement and stem of the implant were removed transabdominally (Figure 2). The general surgeon closed the peritoneal cavity and the abdominal wound in a typical manner, leaving drainage in place for 48 h.

The orthopedic team then proceeded with their part of the procedure. The endoprosthesis stem was removed with the cement from the medullary cavity of the femur. The presence of a Paprosky Type IIIB acetabular defect was confirmed [16]. The defect had the characteristic appearance of a hole involving both the anterior and posterior pelvic column where they passed across the central part of the acetabulum. At the same time, the diameter of the defect was only approx. 4 mm larger than that of the implant head (26 mm, Femoral Head of ULTIMA Total Hip System, Johnson&Johnson). The defect was partially covered with fibrous tissue. The remains of the bony bedding of the acetabulum were debrided. The defect of the acetabulum was filled with allogenic bone grafts—both solid and chopped (frozen head and small grafts of spongy bone) obtaining final closure of the channel penetrating to inner pelvis. During repair of the defect it was found that the bony acetabulum, though thinned, showed linear cracks radiating away from the defect towards the edges. It looked similar to a hinge mechanism of a door opening only to one side, i.e., towards the abdominal cavity. Protrusion of the acetabulum with the head and neck of the implant was possible, but bending the bony membranes in the opposite direction to enable reduction of the implant head was not possible. The pathological anatomical relations were additionally supported by numerous membranous soft-tissue adhesions. The orthopedic team filled the defect in the edge of the acetabulum with a solid bone graft fixed with three Kirschner wires down to iliac bone. The acetabulum was strengthened with a metal mesh. A new prosthesis socket was placed on bone cement (Exeter, Stryker). This was followed by implantation of the implant stem on the cement and the placement of a metal head. The joint was intraoperatively reduced repositioned presenting good static and dynamic stability. A suction drain was placed for 48 h.

Intraoperative blood loss was approximately 900 mL. Post-operative drainage produced 520 mL of blood over the 48 h. A total of 4 units of RBC concentrate and 2 units of plasma were transfused. Antibiotics (Cefazolin 1 g tid for 8 days) and antithrombotic prophylaxis were administered according to established standards.

Rehabilitation was commenced immediately after the surgery (respiratory exercises), with the patient assuming the vertical position directly after removal of the drains, i.e., on the second post-operative day. At the same time, the patient, assisted by a physiotherapist, commenced gait re-education using elbow crutches with partial loading of the operated lower limb. Climbing stairs was taught from post-operative day 6 onwards. The patient was discharged on the 10th post-operative day, with instructions to continue orthopedic treatment and rehabilitation at an outpatient facility. The surgical wound healed without complications. Cultures were negative. Sutures were removed on the 10th post-operative day. At discharge, the patient was able to move about unassisted with elbow crutches. The rehabilitation was made difficult by advanced osteoarthritis of the left hip secondary to protrusion in the course of Otto-Chrobak disease.

The patient reported for a follow-up examination at 6 weeks after the surgery and then at 3 months, 6 months and 12 months. Her opinion of the revision was very good and in October 2008 she underwent an arthroplasty of the left hip because of the osteoarthritis. The surgery and post-operative course were uneventful. Because of this second operation, follow-up examinations in the second year following the revision arthroplasty were more frequent in accordance with the follow-up algorithm for the left hip joint.

Subsequent follow-up visits have taken place regularly once a year. At the most recent stationary follow-up visit in 2013, i.e., six years after the revision surgery, the patient reported no subjective complaints regarding the operated hip joints, in particular the right hip joint. The ranges of motion were as follows: flexion of 100°, hyperextension of 0°, adduction of 10°, abduction of 15°, external rotation of 15°, and internal rotation of 5°. Her HHS score was 83.7 and her NRS score was one.

**Figure 3:**
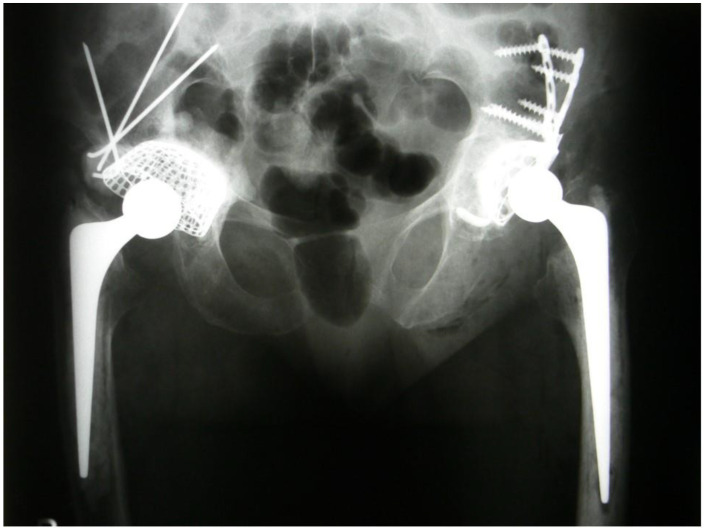
Status post revision arthroplasty of the right hip joint and primary arthroplasty of the left hip joint. An AP radiograph of the pelvis and the hip joints.

Radiographs taken at successive follow-up visits showed correct positioning of the components of the implant and progressive remodeling of the implanted allogeneic grafts. There was no implant migration or loosening at 6 years post-surgery (Figure 3).

Orthopedic team maintained constant telephone contact with patient, due to her residence at a considerable distance from hospital (the last tele-consultation was conducted in February 2020). According to the information received this way, she was satisfied with results of the operation, suffering no any pain neither discomfort in the right hip or abdomen. Her general mobility remained at a good level.

**Figure 4:**
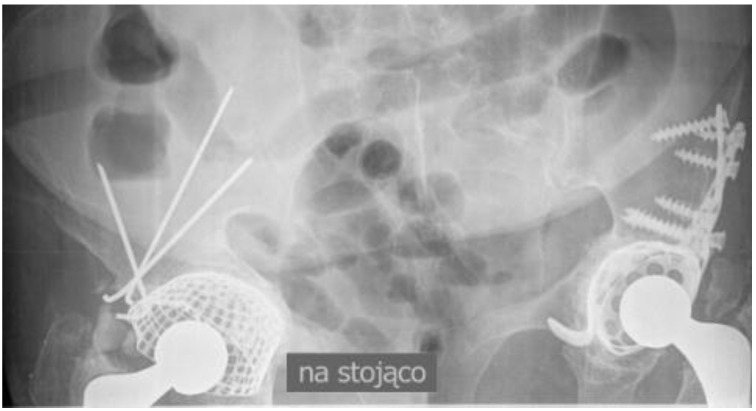
An X-ray of the abdomen visualizing both hip joints 13 years following successful right hip revision arthroplasty.

Thirteen years after the revision right hip procedure, the patient was hospitalized in internal medicine department due to gastroenteritis. During hospitalization, an X-ray of the abdomen was made visualizing both hip joints (Figure 4). Analyzing this radiogram, particular attention should be paid to the perfect remodeling of bone grafts used in the profound compartment of the acetabulum and in its roof with significant improvement of radiological density of the bones surrounding right hip joint.

## 4. Discussion

In a small proportion of cases, not all of which have probably been described in the literature, acetabular loosening and migration leads to the protrusion of the implant acetabulum into the pelvic cavity. Except for cases of trace protrusion, there is always a risk of damage to anatomic structures in the pelvic cavity caused by the migrating acetabular component, cement, or even the implant head and stem [8,9,15]. This is typically seen in acetabular defects classified as Type III B according to Paprosky [16]. Within the pelvic cavity, directly above the bony acetabulum of the hip joint, i.e., over the pelvic bone at the junction of its three components (ilium, pubis, and ischium) is the iliopsoas muscle or, more specifically, a part of it called the iliac muscle. Together with the fascia, the iliac muscle is, biomechanically, the ultimate defensive structure preventing the displacement of a protruding implant deep into the abdominal cavity and damage to vitally important organs. A similar role is played by the greater and lesser trochanter and the intertrochanteric crest, which, resting on the structures of the external surface of the acetabulum, mechanically limit further migration of the stem and head of the femur into the pelvic cavity. An additional defense mechanism involves pain reflexes in the well-innervated area of subperitoneal space. Still, subperitoneal space does house anatomic structures damage which is a threat to the patient’s health or life, most importantly blood vessels, nerves and a ureter. Damage to the internal or external iliac artery, their ramifications or the accompanying veins may be a life-threatening emergency [17,18,19,20,21,22].

When the acetabulum has protruded into the pelvic cavity, the possibility of the above emergencies explains why a revision hip arthroplasty, which in these circumstances is actually life-saving surgery, has to be performed. When it is not possible to retrieve the entire implant with cement from a limb approach, additional access from the abdominal cavity becomes necessary. A typical approach, recommended in the literature, is an incision along the iliac crest (Letournel-Judet). This is followed by the dissection of the iliac muscle while protecting the integrity of subperitoneal space structures. Importantly, this technique does not involve opening the peritoneal cavity [17,19,23,24].

In our patient, the local condition precluded the use of this technique. The consulting general surgeon was not certain whether the iliac muscle could be safely detached from the iliac ala and, consequently, decided to gain access via the peritoneal cavity. The surgeon thought that sharp fragments of the bone cement might dislocate during the dissection of the muscle and perforate the parietal peritoneum into the peritoneal cavity, causing complications, including bowel injury. Consequently, he decided to reach the prosthesis via the peritoneal cavity rather than the extraperitoneal space. The detachment of the iliac muscle, which covers the internal surface of the iliac ala always involves a risk of incidental perforation of the parietal peritoneum by a sharp-edged fragment of the bone cement below the peritoneum and damage to the alimentary tube, vessels or other significant anatomic structures. This is confirmed by the literature [9,25,26,27]. In our patient, when the iliac muscle, covering the superior aspect of the acetabulum, was reached via the peritoneal cavity and it was found that there were no complications already present (the gut was not damaged and integrity of the digestive tract was preserved) and that fragments of cement were placed directly below the parietal peritoneum and caused peritoneal irritation, it was decided that the hip joint reconstruction would proceed as one-stage surgery. In our opinion, if there had been damage to the alimentary tube, it would have been necessary to divide the operative treatment between two or three procedures. The first stage would involve removal the endoprosthesis together with bone cement and the placement of a stoma (or reconstruction of the digestive tract). The second stage would consist in the general surgery procedure of reconstruction of the alimentary tract (if a stoma had been placed in the first stage). Reconstruction of the hip joint, which we performed, would only have been possible in the third stage. These considerations show that, as the risk of damage applies mainly to structures within the abdominal cavity, the decision as to which approach (transperitoneal vs. subperitoneal) to use in order to gain access to the endoprosthesis should be taken by a general surgeon, as was the case with our patient. If there is contamination of musculoskeletal structures with digestive tract bacteria or if even minor collections of pus are present, surgery needs to be divided into stages. The literature points out that a considerable percentage of cases of implant loosening with protrusion of the acetabulum into subperitoneal space are septic and pus collections are present. Luckily, this was not the case with our patient. Otherwise, we would have had to temporarily place a spacer or carry out a Girddlestone procedure. Some papers clearly emphasize the need of a two-stage procedure in the case of septic acetabular loosening with protrusion, as discussed by Stiehl [15]. We fully subscribe to this point of view.

In our patient, there was a risk of injury to anatomic structures in subperitoneal space. In order to reduce this risk, it was necessary to identify anatomical relations in the operated area pre-operatively; therefore. the patient underwent an arteriography. Pre-operative evaluation of the location of vessels and any dislocation is recommended by most relevant papers and we wholly support this recommendation [8,9,15,17]. The examination revealed minor displacement of the vessels anatomically adjacent to the bony acetabulum of the pelvis, which was subsequently confirmed intraoperatively. The ureter was also exposed intraoperatively and found to have intact walls.

Complications reported in the literature include osteolytic destruction of the acetabulum and periacetabular area of the bony pelvis, lesions of the iliac muscle and peritoneum, damage to the femoral nerve, various types of lesions of the iliac vessels and their ramifications, ureter and the urinary bladder, damage to the alimentary tube or even sepsis [8,9,15,17,28,29,30].

A review of the relevant literature revealed significant terminological discrepancies, which proves the complexity, but indirectly also an anecdotal character (noted only in case reports) of the problem. Some papers use the term retroperitoneal to describe the approach to an implant acetabulum that has migrated towards the abdominal cavity [17,24]. This is not the right term to use. Retroperitoneal space is part of extraperitoneal space. Extraperitoneal space is contained between the abdominal walls and the parietal peritoneum. Apart from retroperitoneal space, it includes pre- and subperitoneal space. It is via subperitoneal space that an extraperitoneal approach to expose the acetabulum is made. Subperitoneal space is bounded superiorly by the parietal peritoneum, inferiorly by the pelvic diaphragm and urogenital diaphragm, and is continuous with the retroperitoneal space superoposteriorly. In women, the parietal peritoneum in the pelvic reaches the apex of the urinary bladder anteriorly and covers the posterior surface of the bladder to the level of the ureteral orifices, fills the uterovaginal depression, reaches the uterus at the isthmus, covers the anterior surface of the uterine body, fundus, posterior surface of the body, posterior surface of the supravaginal part of the uterine cervix, posterior vaginal fornix, and reaches the rectum at the level of the Kohlrausch fold to subsequently reach structures of retroperitoneal space. Hence, it should be pointed out that when the implant acetabulum is removed via the abdominal cavity, the structures involved lie in subperitoneal, rather than retroperitoneal, space.

The most important issue that merits some discussion in the case of our patient appears to be the cause of the difficulty in removing the implant acetabulum with bone cement and the implant head and stem, which had followed the acetabular component into the pelvic cavity. It is a complex problem, noted and described by numerous authors. Eftekhar et al. [17] points out the possibility of adhesion formation between vessels and other soft tissue and the cement, which may lead to severe complications if bits of the dislocated cement are removed without proper care. He also notes that giant cells and histiocytes are present in the area and that there is also an extensive scar, which makes it even more difficult to distinguish anatomical structures correctly. Grigoris et al. [24] writes about the importance of numerous adhesions as careless traction on these adhesions may lead to damage to vitally important structures: the destruction of vessels and organs in the pelvic cavity [26,31,32]. Ahmad et al. [8] also underlines the importance of existing adhesions, scar fibrous tissue, and the risk of bleeding associated with an inappropriate attempt to recover the dislocated implant. Girard et al. [19] also emphasizes the need to appropriately protect delicate soft-tissue structures. Tazawa et al. [9], in an analysis of earlier papers by other authors, advocates a transperitoneal approach as a way to prevent complications. We believe that the key to this problem lies in the universally adopted, and regarded as standard, division of acetabular defects according to Paprosky [16]. Type III B in his classification refers to cases of extensive destruction of the acetabulum and disruption of the ring with significant protrusion of the prosthetic acetabulum into the pelvic cavity. This designation actually embraces various types of defects. One, often noticed intraoperatively, is a very extensive defect of the acetabular fossa enabling the protrusion of the implant acetabulum with the head and stem into the pelvic cavity. In such cases, the prosthetic head and stem can be driven out of the pelvic cavity if an appropriate force is applied along the limb axis. However, there is also another type of defect, a less extensive one involving a valve mechanism, that is also classified as Paprosky’s Type III B. This latter presentation was the one seen in our patient. The defect in the bony acetabular fundus was large enough that, combined with additional radiating fracture lines in the remaining part of the roof, elements of the implant could penetrate into the pelvic cavity, while at the same time, a valve-like mechanism made it impossible to drive the acetabulum out of the pelvic cavity by traction or attempts to dislocate it, as that would require a larger defect in the acetabular roof. It follows then that in some cases a larger defect of the acetabulum enables easier reduction from subperitoneal space of the implant head and proximal part of the stem. If this is the case, safe recovery of the implant acetabulum via the lower limb can be attempted. In view of these observations, we believe that it is advisable to further divide Paprosky Type III B acetabular defects into two subtypes. One subtype involves more acetabular destruction and is seemingly more difficult to reconstruct operatively, but actually enables revision surgery to be carried out via the lower limb. The other subtype involves less acetabular destruction but, because of the presence of a valve-like mechanism, requires additional surgical access via the abdominal cavity. In the case of our patient, the defect in the bony acetabulum was relatively small, but large enough to allow migration of the implant. At the same time, it precluded reverse movement of the implant head and stem in the typical manner of intraoperative dislocation. The prosthetic acetabulum together with the bone cement had been displaced into subperitoneal space between iliac muscle structures. Sharp fragments of the cement reached the parietal peritoneum. The head of the femoral bone on the stem had also migrated into the pelvic cavity together with the implant acetabulum. Any maneuvers would have necessarily caused multidirectional movement of the acetabulum on the head and stem together with sharp fragments of the cement, which could have led to injuries to vessels, nerves, ureter, and possibly also the parietal peritoneum and structures of the alimentary tube. Maneuvering was made additionally difficult by membranous soft-tissue structures, described also by other authors, which covered, and crossed in many planes, elements of the implant and the native acetabulum. The operator performing the revision surgery had carried out several dozen revision arthroplasty procedures (according to data from the endoprosthesis register of the National Health Fund). Both the operator and the other authors of this case report had seen the two, biomechanically different, subtypes of Paprosky Type III B acetabular roof defects in their surgical career. However, only a few of the several dozen revision arthroplasties of Paprosky Type III B patients involved this problem. In these cases, the anatomic presentation was always similar, necessitating access via the abdominal wall. The case described in the present paper was the most illustrative example, which is why we chose to report on this particular case. Considering this peculiar clinical situation, not previously described in the literature, we consider that the most appropriate way to refer to it is by comparing it to a valve mechanism, although the membranous soft-tissue structures that were present in our patient and have also been described by other authors may bring to mind a trapped hernia.

For several decades the number of total hip arthroplasty procedures performed around the world has been constantly growing, resulting in an increasing need for effective treatment of various intra- and postoperative complications, including intra-pelvic cup protrusion. It must be remembered, that the surgical technique used in such cases has been changing over the years, along with the development of technology and the acquisition of surgical experience. Today there is a number of reconstructional options available to surgeons that were far less widespread and available ten or fifteen years ago, and the effectiveness of their use was just being investigated and under debate at that time. In the case we are discussing, the surgical technique used allowed to obtain a good, long-term clinical effect (to our knowledge this is the first such long observation in the literature). Analyzing this case after many years, considering great improvement of the surgical skills and easy access to special devices like extended anti-protrusion solid cages fixed with the screws to a healthy bone surrounding defected hip acetabulum, we still confirm high practical utility of the surgical technique we used. We especially propose to consider its disposal in the absence of access to complex and expensive reconstructive implants [19,33].

## 5. Conclusions

To conclude, a revision arthroplasty of the hip joint with an additional access site via the abdominal cavity is an optimal method of managing acetabular protrusion into the pelvic cavity that cannot be managed by conventional reduction. In case of doubt regarding the possibility of safely detaching the subperitoneal space muscles, a transperitoneal approach appears safe and fully justified. On account of differences in the biomechanics of reduction of the implant dislocated into subperitoneal space, we propose to divide Type III B acetabular defects according to Paprosky into two subtypes.

## Data Availability

Data available in a publicly accessible repository.
